# Moderate Treadmill Exercise Alleviates NAFLD by Regulating the Biogenesis and Autophagy of Lipid Droplet

**DOI:** 10.3390/nu14224910

**Published:** 2022-11-20

**Authors:** Yangjun Yang, Xi Li, Zonghan Liu, Xinyu Ruan, Huihui Wang, Qiang Zhang, Lu Cao, Luchen Song, Yinghong Chen, Yi Sun

**Affiliations:** 1 Key Laboratory of Adolescent Health Assessment and Exercise Intervention of Ministry of Education, East China Normal University, Shanghai 200241, China; 2 College of Physical Education and Health, East China Normal University, Shanghai 200241, China

**Keywords:** NAFLD, exercise, lipid droplet, lipophagy, lipid droplet biogenesis, lipid droplet expansion

## Abstract

Lipid droplet is a dynamic organelle that undergoes periods of biogenesis and degradation under environmental stimuli. The excessive accumulation of lipid droplets is the major characteristic of non-alcoholic fatty liver disease (NAFLD). Moderate aerobic exercise is a powerful intervention protecting against the progress of NAFLD. However, its impact on lipid droplet dynamics remains ambiguous. Mice were fed with 15 weeks of high-fat diet in order to induce NAFLD. Meanwhile, the mice performed 15 weeks of treadmill exercise. Our results showed that 15 weeks of regular moderate treadmill exercise alleviated obesity, insulin intolerance, hyperlipidemia, and hyperglycemia induced by HFD. Importantly, exercise improved histological phenotypes of NAFLD, including hepatic steatosis, inflammation, and locular ballooning, as well as prevented liver fat deposition and liver injury induced by HFD. Exercise reduced hepatic lipid droplet size, and moreover, it reduced PLIN2 protein level and increased PLIN3 protein level in the liver of HFD mice. Interestingly, our results showed that exercise did not significantly affect the gene expressions of *DGAT1*, *DGAT2*, or *SEIPIN*, which were involved in TG synthesis. However, it did reduce the expressions of FITM2, *CIDEA*, and *FSP27*, which were major involved in lipid droplet growth and budding, and lipid droplet expansion. In addition, exercise reduced ATGL protein level in HFD mice, and regulated lipophagy-related markers, including increasing ATG5, LAMP1, LAMP2, LAL, and CTSD, decreasing LC3II/I and p62, and promoting colocalization of LAMP1 with LDs. In summary, our data suggested that 15 weeks of moderate treadmill exercise was beneficial for regulating liver lipid droplet dynamics in HFD mice by inhibiting abnormal lipid droplets expansion and enhancing clearance of lipid droplets by lysosomes during the lipophagic process, which might provide highly flexible turnover for lipid mobilization and metabolism. Abbreviations: β-actin: actin beta; ATG5: autophagy related 5; LAMP2: lysosomal-associated membrane protein 2; LAMP1: lysosomal-associated membrane protein 1; SQSTM1/p62: sequestosome 1; GAPDH: glyceraldehyde-3-phosphate dehydrogenase; MAP1LC3/LC3: microtubule associated protein 1 light chain 3; ATGL: adipose triglyceride lipase; CSTD: cathepsin D; LAL: lysosomal acid lipase; DGAT1: diacylglycerol-o-acyltransferase 1; DGAT2: diacylglycerol-o-acyltransferase 2; CIDEA: cell death inducing dffa-like effector a; CIDEC/FSP27: cell death inducing dffa-like effector c; FITM2: fat storage-inducing transmembrane protein 2; PLIN2: adipose differentiation related protein; PLN3: tail-interacting protein 47; HSP90: heat shock protein 90; SREBP1c: sterol regulatory element binding protein-1c; chREBP: carbohydrate response element binding protein.

## 1. Introduction

Non-alcoholic fatty liver disease (NAFLD) has emerged as the primary cause of chronic liver diseases worldwide, with an estimated global prevalence of 25% [1]. NAFLD patients are characterized by progressive hepatic steatosis, inflammation, locular ballooning, cellular damage, and fibrosis, a process that may take many years to proceed. It is well known that the vast majority of NAFLD cases arise from abnormal accumulation of lipid droplets (LDs), especially large-size LDs. [2]. As highly dynamic intracellular depots, LDs alternate between periods of biogenesis (triacylglycerol synthesis, growth, budding, and expansion of LDs) and degradation (lipolysis and lipophagy) [3]. This dynamic process can be regulated by several stimuli, such as high-fat diet, starvation, lack of energy, and hypoxia [4,5]. Although the precise mechanism of LD dynamics is not entirely clear yet, mounting evidence suggests that dysregulated LD biogenesis and degradation lead to the abnormal accumulation of LDs and contribute to the development of lipotoxicity, chronic hepatic insulin resistance, and inflammation [6]. In addition to DGAT2, a triglyceride synthase, other lipogenic-related transcription factors such as SREBP1c and chREBP, as well as lipid droplet fusion-related genes were also highly expressed in the liver of HFD-induced NAFLD mice [7,8]. Notably, inhibiting lipophagy pharmacologically or genetically led to an increase in hepatic lipid content and lipid droplet sizes [9,10]. Moreover, abnormal accumulation of lipolysosomes in liver biopsies was also shown in NAFLD patients [11]. Therefore, restoring dysregulated lipid droplet dynamics might provide a therapeutic option to ameliorate NAFLD.

Importantly, an unhealthy lifestyle, which includes factors such as saturated fat-rich diets, cholesterol fat-rich diets, sugared drinks as well as lack of physical activity, has been directly related to the susceptibility of developing NAFLD [12]. Currently, there are no established pharmacological therapies for the treatment of NAFLD without side effects. However, it is well-established that aerobic exercise training is beneficial for preventing and improving NAFLD in both human clinical trials and animal studies [13]. Much of the accumulated evidence in NAFLD rodent models suggested that regular bouts of exercise decreased lipid content and the number and size of hepatic LDs [14]. The most discussed mechanisms are the lipid metabolism of LDs core, including normalizing de novo lipogenesis and lipolysis [15], restoring impaired lipophagy [16], and increasing triglyceride export marker apoB100 [17]. It should be noted that LD dynamics may modify the process of lipid accessibility of the LD core. However, few studies have evaluated the effects of exercise on LDs dynamics (growth, assembly, expansion, and degradation) itself.

Even though regular bouts of aerobic exercise training are considered beneficial for decreasing hepatic lipid content [18], it may not exert the same effect on all phases of NAFLD patients, since the disease status may dictate the outcome of exercise intervention [19]. This phenomenon has also been reported in animal models [15,20]. Of note, there is a particular need for preventive measures in NAFLD patients, since the intra-and extrahepatic benefits obtained from prevention are potentially greater [21]. However, most of the studies analyzing the effects of exercise on preventing NAFLD have been performed during short-term periods (4–8 weeks). There are fewer studies characterizing the potential beneficial actions of long-term exercise in HFD-induced NAFLD mice. Moreover, there has been no systematic research on the impact of exercise on the entire process of LDs dynamics in protecting against the progress of NAFLD.

Therefore, the aim of this study was to investigate the effect of 15 weeks of regular treadmill exercise on preventing NAFLD. Moreover, we aim to characterize the potential mechanism of LD dynamics, including LD biogenesis and degradation. We found that exercise could regulate the size and content of hepatic LDs by inhibiting the abnormal expansion of LDs and restoring lipophagic activity, which might provide a flexible dynamic process for lipid mobilization and metabolism.

## 2. Materials and Methods

### 2.1. Animals

In this case, 24 C57BL/6 male mice (5 weeks of age) were purchased from the animal experiment center of East China Normal University (Shanghai, China). All the mice were housed in a specific pathogen-free room with conventional laboratory conditions (22 ± 2 °C, 40–60% relative humidity) on a 12:12 h light-dark cycle (6 a.m.–6 p.m.), with access to water and food ad libitum. Body weight was measured weekly until the mice were sacrificed.

### 2.2. Diets and Exercise Intervention

**Figure 1:**
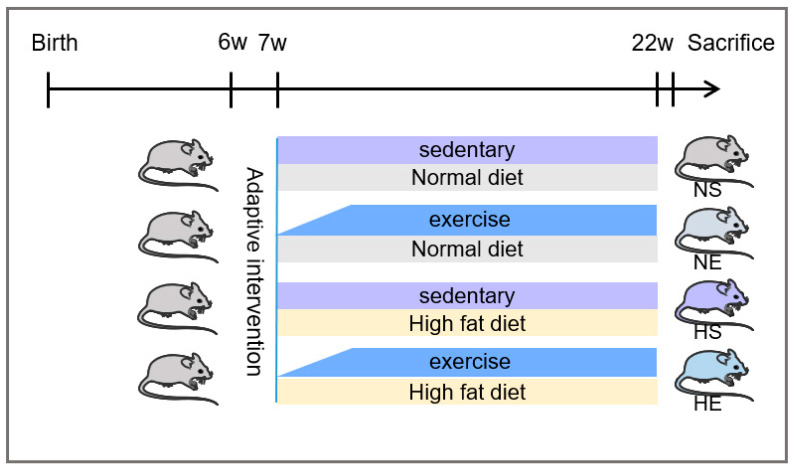
The timeline and animal grouping in the experimental design. All animals were divided into four groups, normal diet sedentary (NS, *n* = 6), normal diet combined exercise (NE, *n* = 6), high-fat diets sedentary (HS, *n* = 6), and high-fat diet combined exercise (HE, *n* = 6). The mice from HFD groups (HS and HE) were fed with 60% fat diets (D12492, Research diets, New Brunswick, NJ, USA) for 15 weeks.

After one week of adapting to the environment, the mice were randomly assigned to the following groups: Normal diet sedentary (NS, *n* = 6), Normal diet combined exercise (NE, *n* = 6), High-fat diets sedentary (HS, *n* = 6), and High-fat diet combined exercise (HE, *n* = 6). The mice from HFD groups (HS and HE) were fed with a high-fat diet contained 60% fat, 20% carbohydrate, and 20% protein in terms of kcal (#D12492, Research diets, New Brunswick, NJ, USA) for 15 weeks (Figure 1).

The treadmill running protocol was adapted from Liu et al. [22]. Mice in Group NE and Group HE received a 5-day adaption period followed by 15 weeks of training.

During the adaption period, the running speed was increased from 7 m/min to 13 m/min, and the running duration was increased from 15 min/day to 60 min/day. During the formal training period, the mice were subjected to a training protocol during 7–9 a.m., for 60 min/day, and 5 days/week for 15 weeks. Each day, mice were trained in a sequence in the following order: 5 min for warming up (7 m/min), 60 min for formal training (13 m/min), and 5 min for relaxing (7 m/min). Mice from Group NS and Group HS were also placed on the static treadmill for the same duration of time. We avoided using electric shock stimulation in our experiments and used a cork stick to push and drive the mice throughout to reduce their pain. Their body composition (AccuFat-1050, MAG-MED, Jiangsu, China) was analyzed 24 h after the last exercise intervention. The mice were sacrificed 36 h after the last training session, following overnight fasting.

### 2.3. Insulin Resistance Test (ITT) and Biochemical Analysis

One week before the end of the exercise intervention, ITT was carried out. After 4 h of fasting (9:00 a.m. to 1:00 p.m.), blood was collected from the tails for glucose measurement at the time of 0 min, 15 min, 30 min, 60 min, 90 min, and 120 min. After the first blood glucose test (t = 0 min), the mice immediately accepted an injection (intraperitoneal) of insulin (J20050098, Novo Nordisk, Tianjin, China) at 0.5 U/kg body weight.

### 2.4. Serum Biochemical Analysis

The blood samples were centrifuged (5804/R, Eppendorf, Hamburg, Germany) at 3000 r/min for 10 min to acquire the serum. Serum samples were stored at −80 °C. All the assay kits were obtained from Nanjing Jiancheng Bioengineering Institute (Nanjing, China) for serum TC (A111-1-1), LDL-c (A113-1-1), aspartate aminotransferase (AST) (C010-2-1), and alanine aminotransferase (ALT) (C010-2-1).

### 2.5. Histological Analysis

The liver tissues were extracted immediately after harvesting and immersed in 10% neutral buffered formalin for 24 h. One part of these tissues was dehydrated using 70% ethanol, embedded in the paraffin, and cut into a 5 μm coronal section for hematoxylin and eosin. The same liver lobe of each animal was captured by using the Nikon View software (Nikon DS-U3, Tokyo, Japan) (*n* = 3). Evaluation of NAFLD activity score (NAS) has been described before [23]. We receive information for steatosis (<5% = 0, 5–33% = 1, 33–66% = 2, >66% = 3), lobular inflammation (none = 0, <2 foci = 1, 2–4 foci = 2, >4 foci = 3), and locular ballooning (none = 0, few = 1, prominent = 2). The NAS is the unweighted sum of steatosis, lobular inflammation, and hepatocellular ballooning scores.

The other part of these tissues was embedded in tissue-Tek OCT (Sakura Finetek, Torrance, CA, USA) and cut into a 5 μm coronal section for Oil Red O staining. The images were visualized under light microscope (Nikon Eclipse E100, Japan). The number and area of LDs were analyzed with Image J version 1.51 software and Image-pro plus 6.0 (National Institutes of Health, Bethesda, MD, USA). All of these features were scored in a blinded manner per sample.

### 2.6. RNA Extraction and Quantitative RT-PCR

The total RNA was extracted from liver samples (40 mg per sample) by the Trizol method (Invitrogen, Thermo Fisher Scientific, Carlsbad, CA, USA), as per the manufacturer’s instruction. For real-time PCR analysis, reversing transcription of RNA was performed using a cDNA reverse transcription kit (FSQ-101, TOYOBO, Osaka, Japan). Gene expression levels were further detected by using an RT-PCR reaction mixture containing SYBR-green fluorescent dye (11202ES03, Yeasen, Shanghai, China). Primers used are listed in Table 1. 

### 2.7. Western Blot

Western blot was conducted as previously described [24]. Liver tissue (20 mg) was homogenized and lysed with RIPA lysis buffer (0.1% sodium deoxycholate, 0.5% NP-40, 150 mM NaCl, 50 mM Tris-Cl, pH 7.5; P0013B), PMSF (ST506), phosphatases and protease inhibitors (P1045). In brief, the liver tissue was loaded in a homogenate tube with 0.4 mL lysis buffer and four magnetic beads and mechanically homogenized at 3.55 m/s for 30 s. The homogenization was repeated three times, and further centrifuged at 4 °C for 10 min at 12,000× *g*. The supernatant was collected. Bradford method (P0010S) was used to determine the protein concentration of total homogenate.

The samples (25 µg) were separated on 10–12% SDS-PAGE gel and transferred to the PVDF membrane (Millipore). The membranes were then blocked in QuickBlock™ Blocking Buffer for Western Blot (P0252, Beyotimebio, Shanghai, China) for 1.5 h at room temperature and incubated with the following primary antibodies: ADRP/perilipin2 (15294-1-AP), LC3B (14600-1-AP), lysosomal acid lipase (LAL)(12956-1-AP), and Cathepsin D (CTSD)(21327-1-AP) were obtained from Proteintech (Wuhan, China), adipose triglyceride lipase (ATGL)(#2439), Beclin1 (#3495S), ATG5 (#12994), and LAMP1 (#3243) were obtained from Cell signaling technology (Danvers, MA, USA), TIP47/PLIN3 (sc-390968, Santa Cruz, CA, USA), anti-sqstm1/p62 (ab109012, Abcam, Cambridge, MA, USA), LAMP2 (DF6719, Affinity, Jiangsu, China), and horseradish peroxidase-conjugated secondary antibodies (115-035-003, 111-035-003, Jackson Immunoresearch, West Grove, PA, USA). The images of protein bands were captured and analyzed by the ChemiDoc MP Imaging System (BIORAD, California, CA, USA). The β-actin (sc-8432, Santa Cruz, CA, USA), HSP90 (ab178854, Abcam), alpha-tubulin (AF7010, Affinity), and GAPDH (GB12002, Servicebio, Wuhan, China) were used to normalize the amount of protein loaded. Unless otherwise specified, all biochemical reagents were purchased from Beyotime, Shanghai, China.

### 2.8. Immunofluorescence Staining

The paraffin-embedded liver tissue sections were cut into 10 μm coronal sections and used for immunofluorescence staining. These prepared sections were blocked with mouse serum and incubated with ADRP/perilipin2 (15294-1-AP, Proteintech, Wuhan) overnight at 4 °C, followed by fluorescence conjugated secondary antibody (Servicebio, Wuhan, China). The sections then added CY3-TSA (Servicebio, Wuhan) and were incubated in dark for 10min, followed by antigen retrieval with EDTA antigen retrieval buffer (1 mM EDTA, pH 8.0). Subsequently, these sections were incubated with the second primary antibody LAMP1 (ab208943, abcam), and followed by a fluorescence conjugated secondary antibody from Servicebio. The immunofluorescence was captured by using a Nikon Eclipse C1 microscope.

### 2.9. Statistical Analysis

Statistical analysis was performed using GraphPad Prism (version 8.0, La Jolla, CA, USA). A two-way analysis of variance (ANOVA) with Turkey methods (with high-fat diet and exercise intervention as factors) was used for the evaluation of differences among the four groups. ITT data were analyzed using repeated-measures ANOVA. The Pearson correlation coefficient was used to calculate correlation coefficient. The statistical significance was considered at *p*  <  0.05. All descriptive values are reported as means ± SEM.

## 3. Results

### 3.1. Exercise Training Alleviates the NALFD-Related Risk Factors in HFD Mice

**Figure 2:**
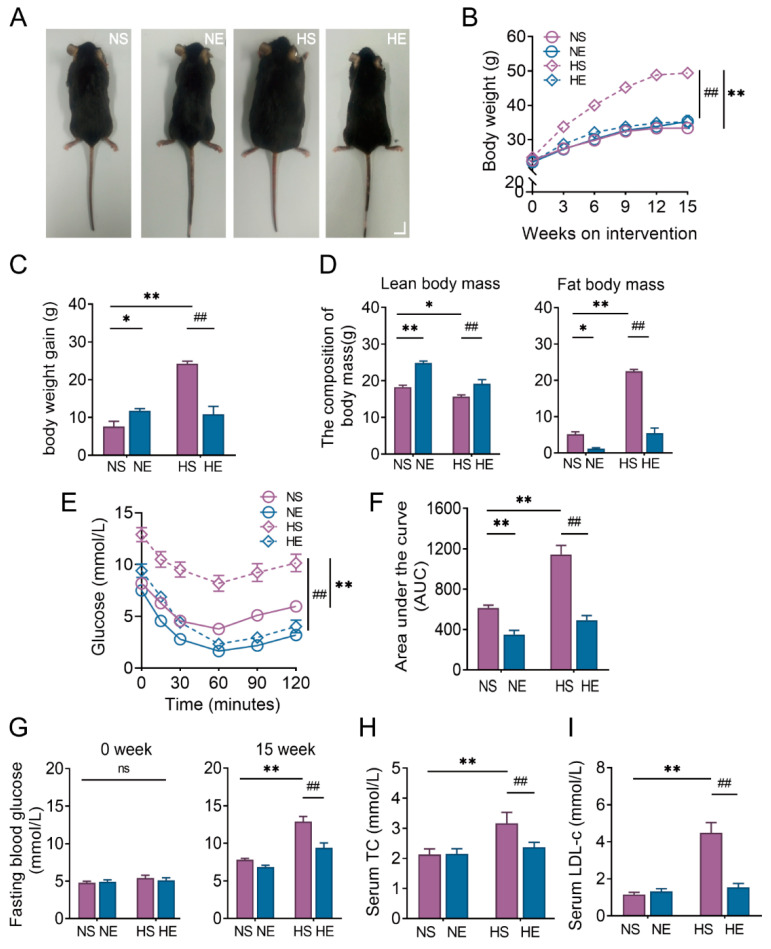
Exercise training alleviates the NALFD-related risk factors in HFD mice. The beneficial effect of exercise in preventing NAFLD was shown by (**A**) the appearance of mice after 15 weeks of intervention (scale bar: 10 mm), (**B**,**C**) body weight and gain of mice, (**D**) body composition, (**E**,**F**) insulin sensitivity tested by ITT, (**G**) fasting blood glucose before and after the intervention, and (**H**,**I**) serum TC and LDL-c. Unless otherwise specified, six mice per group were used in each experiment. The data are expressed as mean ± SEM. * *p* < 0.05, ** *p* < 0.01 versus NS mice; ## *p* < 0.01 versus HS mice.

Poor dietary habits are highly associated with the development of NAFLD [25]. In our study, the mice were fed with HFD for 15 weeks to recapitulate NAFLD [26] (Figure 2A). Their body weights were measured weekly. As shown in Figure 2B, HFD significantly increased body weight in mice, while exercise alleviated the body weight gain of mice induced by HFD, which was associated with reduced fat mass content and increased lean mass content (Figure 2C,D).

NAFLD, recognized as a hepatic manifestation of metabolic syndrome, is usually accompanied by insulin resistance, hyperglycemia, and dyslipidemia as background factors [27]. We first conducted i.p. ITT and observed that exercise significantly improved insulin sensitivity in both HFD and ND-fed mice. Consistently, exercise was able to reduce the elevated levels of fasting blood glucose, TC, and LDL-c of mice induced by HFD (Figure 2G–I).

### 3.2. Exercise Training Suppresses the Development of NAFLD in HFD Mice

**Figure 3:**
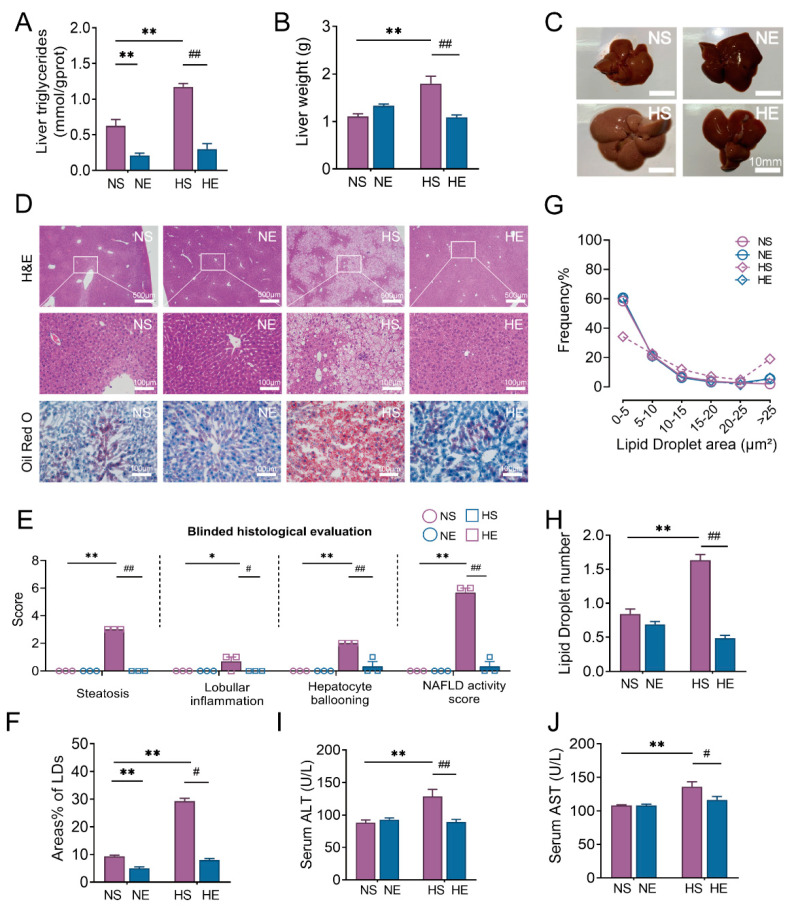
Exercise training suppresses the development of NAFLD in HFD mice. (**A**,**B**) Liver weight and triglycerides. (**C**,**D**) Representative macroscopic morphology, H&E, and Oil red O staining in the liver from each group (scar bar: 10 mm for macroscopic morphology, scar bar: 500 μm and 100 μm for H&E staining, scar bar: 100 μm for Oil red O staining) (*n* = 3). (**E**–**H**) The analysis of NAFLD activity score and Oil red O staining (*n* = 3). (**I,J**) serum ALT and AST. Unless otherwise specified, six mice per group were used in each experiment. The data are expressed as mean ± SEM. * *p* < 0.05, ** *p* < 0.01 versus NS mice; # *p* < 0.05, ## *p* < 0.01 versus HS mice.

Abnormal hepatic LDs deposition is the main characteristic of NAFLD, which may further lead to liver injury [28]. In this study, we observed that HFD induced a fatty color change in liver, and increased liver weight and triglyceride content, whereas exercise significantly reversed the above pathological changes in HFD mice (Figure 3A–C).

Histological examination of liver tissues revealed that exercise significantly reduced the score of hepatic steatosis, inflammation, locular ballooning, and NAFLD activity in HFD mice (Figure 3D,E). The hepatic LDs were stained with Oil Red O, and the result was shown in Figure 3F–H. Consistent with the above results showed, exercise significantly decreased the number and area of hepatic LDs in HFD mice, indicating the effect of exercise in preventing aberrant LDs deposition (Figure 3F). Furthermore, exercise reversed the elevated levels of serum AST and ALT in HFD mice (Figure 3G,H).

### 3.3. Exercise Inhibits LD Biogenesis by Preventing LD Expansion

As the above results showed, exercise is sufficient to decrease the accumulation of hepatic LDs in HFD mice. With this in mind, we next investigated the molecular mechanism of exercise in regulating hepatic LDs dynamics. In general, LDs accumulation is coordinated by transformation of LD membrane proteins and accumulation of lipid core [29].

**Figure 4:**
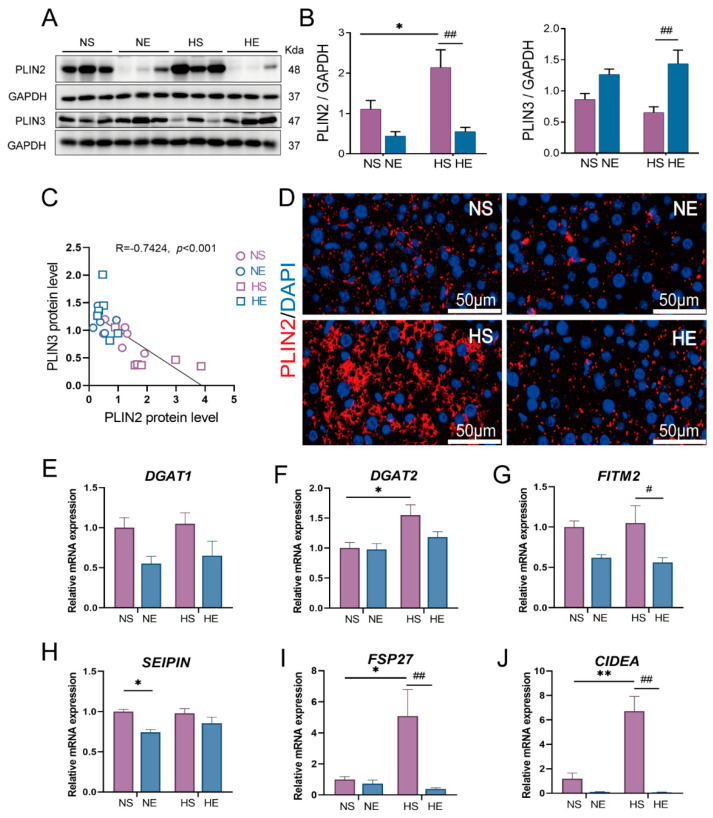
Exercise inhibits LD biogenesis by preventing LD expansion. (**A**,**B**) Representative of LD membrane protein PLIN2 and PLIN3 immunoblotting and quantified data, (**C**) analysis of the correlation between protein levels of PLIN2 and PLIN3 (*n* = 6), (**D**) representative immunofluorescence of PLIN2 in liver tissue (scar bar: 50 μm, *n* = 3), (**E**–**J**) expression of LD biogenesis gene for DGAT1, DGAT2, FITM2, SEIPIN, CIDEA, and CIDEC were measured. Gene GAPDH was used as an internal reference gene (*n* = 5). The data are expressed as mean ± SEM. * *p* < 0.05, ** *p* < 0.01 versus NS mice; # *p* < 0.05, ## *p* < 0.01 versus HS mice.

As shown in Figure 4A, protein levels of PLIN2 and PLIN3, which are involved in regulating the structure and function of different stages of LD biogenesis, were measured. We observed that HFD induced a significant increase in PLIN2 protein level, whereas exercise significantly reduced the PLIN2 protein level in HFD mice. Interestingly, the PLIN3 protein level was not affected by HFD, and however, was increased by exercise intervention (Figure 4A). We further assessed the correlation between the PLIN2 and PLIN3 protein levels, and found that there was a significantly inversely correlation between these two protein levels (Figure 4B). Given that PLIN2 surrounds the LDs of varying sizes, immunofluorescence staining was performed to evaluate hepatic PLIN2 [30]. Our results showed that the large size of PLIN2-positive vesicles (The hollow part encapsulated by PLIN2 is the lipid core) induced by HFD was alleviated by exercise intervention (Figure 4C).

In order to investigate the mechanism that exercise regulated hepatic LDs biogenesis, the expression levels of genes involved in controlling triglycerides synthesis, LD growth and budding, and LD expansion, including DGAT1, DGAT2, FITM2, SEIPIN, CIDEA, and FSP27, were examined (Figure 4D). The results showed that the expression of triglyceride synthesis enzyme DGAT2 and LD fusion genes, such as FSP27 and CIDEA, was significantly increased in the liver of HFD mice. On the contrary, exercise markedly decreased the mRNA expressions of FITM2, FSP27, and CIDEA in HFD mice. These results suggested that exercise might regulate LDs quality by inhibiting LDs expansion instead of the TG synthesis.

### 3.4. Exercise Enhances the Degradation of Hepatic LDs in Lysosomes of HFD Mice

**Figure 5:**
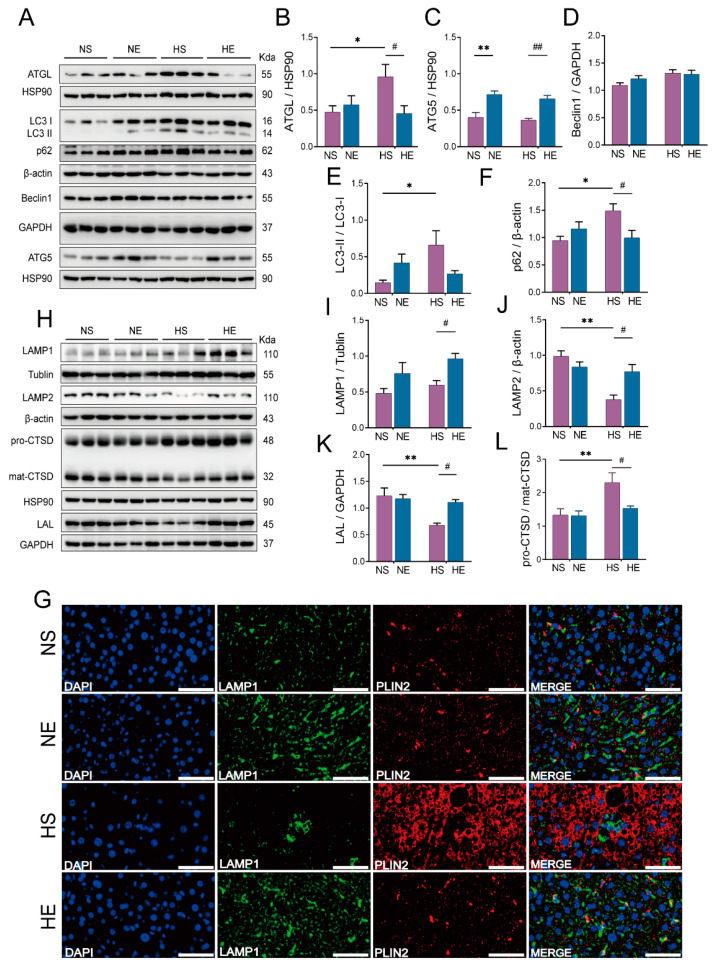
Exercise enhances the degradation of hepatic LDs in lysosomes of HFD mice. (**A**–**F**) Representative of autophagy-associated proteins immunoblotting and quantified data, including ATGL, Beclin1, LC3B, ATG5, and p62, (**G**) representative of lipophagy-related immunofluorescence by the colocalization of PLIN2 and LAMP1 in the liver (scar bar: 50 um, *n* = 3), (**H**–**L**) representative of lipophagy-associated lysosomal proteins immunoblotting and quantified data, including LAMP1, LAMP2, LAL, and CTSD. Unless otherwise specified, six mice per group were used in each experiment. The data are expressed as mean ± SEM. * *p* < 0.05, ** *p* < 0.01 versus NS mice; # *p* < 0.05, ## *p* < 0.01 versus HS mice.

Lipolysis and lipophagy are two major forms of LD degradation within cells. In order to evaluate the degradation pathway of LDs, we first measured ATGL, the key enzyme in the first step of neutral lipid lipolysis [31]. Our result showed that HFD induced an increase in ATGL protein level, while exercise reversed the elevated ATGL in HFD mice (Figure 5B). We further investigated hepatic autophagic activity. The protein levels of LC3II/I, Atg5, p62, and beclin1, were examined. The results showed that HFD induced an increase in protein levels of LC3-II/I and p62. On the contrary, exercise significantly decreased the p62 protein level, and moreover, increased the Atg5 protein level in HFD mice, whereas it only slightly decreased the elevated LC3II/I induced by HFD (Figure 5C–F).

Notably, lipophagy is a special form of autophagy. During this process, LDs were sequestered in autophagosomes (lipophagosomes) for degradation in lysosomes. In general, the LD sequestered in the lysosome is a symbol of lipophagy [16]. Here, the immunofluorescence technique was used to evaluate the colocalization of LAMP1 and PLIN2, which are the substitute for lysosomes and LDs, respectively [32]. The result showed that HFD inhibited the colocalization of PLIN2 with LAMP1, and exercise promoted the colocalization of PLIN2 and LAMP1, suggesting that exercise might have the ability in restoring lipophagy in the liver of HFD mice (Figure 5G).

Given that lysosomes are considered as the destination for lipophagy, we further evaluated protein levels of molecules involved in sustaining hepatic lysosomal function, including LAMP1, LAMP2, CTSD, and LAL. The results showed that HFD induced a decrease in protein levels of LAMP2 and LAL, and an inhibition in CTSD maturation (increased pro-CTSD/mature-CTSD). On the contrary, exercise significantly increased the protein levels of LAMP1, LAMP2, and LAL, and decreased the protein levels of pro-CTSD/mature-CTSD in HFD mice (Figure 5H–L).

## 4. Discussion

Our findings demonstrated that LD dynamics was an important hepatic mechanism of exercise benefits during the development of NAFLD. Our data showed that 15 weeks of HFD tilted the balance of LD dynamics towards increased biogenesis (especially LD expansion), and thus resulted in large-size LD accumulation. At the same time, the degradation of LDs was partly inhibited. On the contrary, 15 weeks of moderate treadmill exercise inhibited the unorganized LD expansion and titled the balance of LDs dynamics towards lipid degradation via lipophagy.

Clinical evidence shows that subjects with NAFLD are more likely to have a greater body mass index, higher serum concentrations of total cholesterol, increased fasting glucose level, and insulin resistance [33]. In this study, we used HFD intervention to induce NAFLD in mice. Mice fed with 15 weeks of HFD developed obesity, insulin intolerance, hyperlipidemia, and hyperglycemia (Figure 2A–I). In addition, the HFD mice manifested a histological liver phenotype of NAFLD, including increased liver weight, liver triglycerides, steatosis, inflammation, locular ballooning, and liver damage, recapitulating unhealthy lifestyle-associated NAFLD (Figure 3A–H).

As expected, we observed that 15 weeks of exercise intervention in HFD mice was sufficient for preventing the development of NAFLD, manifested by alleviation of HFD-induced obesity, insulin intolerance, abnormally elevated fasting glucose, serum TC and LDL-c, liver weight, liver triglyceride, NAFLD activity score, and increased liver damage.

It is well known that LD is the majority form of triglyceride storage. Therefore, we further evaluated hepatic LD content. Our results showed that HFD induced an abnormal increase in content, area, and number of hepatic LD, which was ameliorated by 15 weeks of aerobic exercise intervention (Figure 3D–H). The results led us to speculate that LDs dynamics might be a key mechanism mediating the benefits of exercise in NAFLD.

LDs undergo dynamic periods of biogenesis and degradation under different environmental stimuli, such as nutrient deprivation, and hypoxia. The PAT family proteins, including perilipin (PLIN1), ADRP (PLIN2), TIP47 (PLIN3), S3-12 (PLIN4) and OXPAT (PLIN5), have been shown to play an important role in regulating LD dynamics. In mammals, PLIN2, located almost exclusively in LDs, is closely associated with intracellular lipid content, and is highly expressed in the liver of fatty liver patients [34]. PLIN3 (TIP47) is considered a marker of nascent LDs formation and is distributed in the minuscule hepatocellular LDs [35,36]. As expected, our results showed that HFD increased the level of PLIN2, which was decreased by exercise intervention. On the contrary, 15 weeks of HFD did not cause a significant change in PLIN3 level (Figure 4A,B). Moreover, exercise significantly increased the level of PLIN3 in HFD mice. Wolins et al. [37] observed that under oleate stimulation, PLIN3 would be replaced by PLIN2 during nascent LDs biogenesis (enlarge) and mainly coated smaller LDs. Interestingly, in our model, there was a negative correlation between the PLIN2 protein level and PLIN3 protein level (Figure 4C). The results above led us to speculate that 15 weeks of exercise intervention might have induced a remodeling of hepatic LDs size and structure. We further evaluated LDs size by measuring PLIN2 level which has been used as a surrogate marker in the histopathological examination of tissues for different sizes of LDs [34]. As expected, HFD induced an increased size of PLIN2-positive stained circular droplets, which was inhibited by 15 weeks of exercise (Figure 4D). Therefore, combining the previous reports with our results, we concluded that HFD increased LDs size by inducing lipid droplet biogenesis. On the contrary, exercise intervention decreased LDs size and increased number of small-size lipid droplets by regulating lipid droplet dynamics.

To further confirm and understand this phenomenon, we measured the expression of genes involved in several steps of LD biogenesis, such as TG synthesis, LD growth and budding, and expansion of LDs (Figure 4E–J). Consistent with previous studies [38,39], we found that HFD induced a significant increase in gene expression related to TG synthesis (DGAT2) and LD expansion (CIDEA, CIDEC). On the contrary, exercise significantly reduced the expression of LD expansion-related genes FITM2, CIDEA and CIDEC. Consistent with our study, Reynolds [40] showed that 10 weeks of voluntary wheel running was able to reduce the expression of CIDEA and CIDEC in the liver and adipose tissue of HFD mice. These results demonstrated that exercise might have the ability in preventing LD abnormal enlargement of LD by inhibiting LD fusion and expansion.

The change In the pattern of lipid droplet accumulation induced by exercise might provide a higher chance for energy mobilization, since inhibition of unlimited fusion and expansion of LD increases the total surface of the LDs and thereby increases the access of several lipid degradation components to stored lipid [41]. In general, there are two main ways for regulation of LD degradation, those being lipolysis and LD autophagy (lipophagy). In addition, Schott et al. [42] found that ATGL had a higher affinity to larger-size LDs, while lipophagy was restricted to small LDs. Therefore, we further measured the two major LD degradation pathways in liver tissues. Our results found that exercise decreased the elevated protein level of ATGL, the first rate-limiting lipolytic enzyme, in HFD mice (Figure 5A,B). This result led us to speculate that exercise might activate LDs degradation preferentially through lipophagic pathway LD rather than through lipolysis.

Lipophagy exerts physiological functions by eliminating LD via the autophagic core mechanism [43]. In our study, we found that HFD significantly increased LC3II/I ratio, which occurred in parallel with the upregulated level of p62 (Figure 5E,F). It should be noticed that increased LC3-II/I might suggest an increased formation of autophagosome, while the higher level of p62, a selective substrate adaptor of autophagy that should be degraded with cargo, suggested that the autophagic flux was impaired. Indeed, several studies have described that the accumulation of LC3-II could be a consequence of impaired autophagic flow [44]. Our current data showed that exercise slightly decreased the enhanced LC3II/I level in HFD mice, and moreover, significantly decreased the increased p62 protein level in HFD mice (Figure 5E,F). In addition, Atg5, which was involved in autophagosome formation, was significantly increased by exercise intervention in HFD mice (Figure 5C). The results above suggested that 15 weeks of moderate exercise was sufficient to promote autophagosome formation and to restore the impaired autophagic flux induced by HFD.

We, thus, further measured lipophagy by performing immunofluorescence staining. In this case, 15 weeks of exercise intervention was able to restore the HFD-induced impaired colocalization of PLIN2 and LAMP1 (Figure 5G). Consistent with our results, Li et. al [32] also observed that colocalization of hepatic LAMP1 and LD was promoted by 16 weeks of swimming in HFD mice. To our knowledge, only a few studies have investigated the relationship between exercise and lipophagy in the liver. Gao et al. [16] observed that 8-weeks of treadmill exercise promoted autophagic and lipophagic flux in the NAFLD rodent model. Moreover, Li et al., described that 16 week of swimming promoted hepatic lipophagy of HFD mice by activating the AMPK-SIRT1 pathway. However, the main thrust of previous studies was focused on the initiation of lipophagy, while little was known about whether exercise affected the lipophagic-related lysosomal function in NAFLD mice. Given that lysosomes play a key role in LD degradation, we further examined the effects of exercise on clearance of hepatic LD through lysosomes. As expected, 15 weeks of exercise intervention was sufficient to alleviate the decreased LAMP2, a critical determinant of autophagosome-lysosomefusion in HFD mice [45] (Figure 5J). These results were consistent with our immunofluorescence experiment, suggesting that exercise might restore the fusion of lipophagosome and lysosome in HFD mice. Furthermore, lysosomal acidification is an important parameter determining the activity of lysosomal hydrolases and lysosomal function [46]. Our study showed that HFD increased the pro-CTSD/act-CTSD ratio, whereas exercise reversed the elevated ratio of pro-CTSD/act-CTSD in HFD mice (Figure 5L). Consistent with our study, previous studies had demonstrated that the pro-CTSD/act-CTSD ratio was significantly increased in NAFLD mice and cell models of different backgrounds, which was parallel with impaired lysosomal acidification [47]. Of note, even if lysosome contains several hydrolases, lysosomal acid lipase (LAL) is the only lipase in lysosomal that functions by hydrolyzing triglycerides to generate free fatty acids [45]. Baratta et al. [48] observed that the activity of LAL was decreased in NAFLD patients. Previous studies also showed that reversing the expression of LAL in LAL^−/−^ mice by gene or enzyme methods could decrease the abnormal excess lipid storage in the liver [49,50]. In the current study, we observed that HFD decreased the level of LAL, whereas exercise reversed the protein levels of LAL in HFD mice (Figure 5K). Our study also demonstrates the benefit of exercise on improving LD degradation through enhancing lysosomal function (lysosomal-autophagosome fusion, acidification, acid lipase amounts) during the later stages of lipophagy.

## Figures and Tables

**Table 1:** Primers sequence.

Gene		Sequence (5′→3′)
DGAT1	Forward	GCTTGCTTCAGATAGGCTCTTC
	Reverse	ATGGTGCCCAAGCTCAAG
DGAT2	Forward	CGAGACACCATAGACTACTTGCT
	Reverse	GCGGTTCTTCAGGGTGACTG
FITM2	Forward	TCATTGCCCTTACCAACTACCA
	Reverse	AGTGGCCCGAGATGTCAAA
Seipin	Forward	CTGTTGCCAATGTCTCACTGG
	Reverse	TCTAAGGTGACTCGATATGGCTG
CIDEA	Forward	TGACATTCATGGGATTGCAGAC
	Reverse	GGCCAGTTGTGATGACTAAGAC
CIDEC/FSP27	Forward	ATGGACTACGCCATGAAGTCT
	Reverse	CGGTGCTAACACGACAGGG
GAPDH	Forward	AGGTCGGTGTGAACGGATTTG
	Reverse	TGTAGACCATGTAGTTGAGGTCA

## Data Availability

Data and publication materials are available from de corresponding author on a reasonable request.
